# Supplementary material for: Rejuvenation of plasticity via deformation graining in magnesium
Source: Nat Commun. 2022 Feb 25;13:1060. doi: 10.1038/s41467-022-28688-9 (PMC8881527; doi:10.1038/s41467-022-28688-9)
Supplement: Supplementary file 1 — Supplementary Information [file 41467_2022_28688_MOESM1_ESM.pdf]

# Supplementary Information for

# Rejuvenation of plasticity via deformation graining in magnesium

Bo-Yu Liu[1†], Zhen Zhang[2†]*, Fei Liu[1], Nan Yang[1], Bin Li[3], Peng Chen[4], Yu Wang[5], Jin-Hua Peng[2,9], Ju Li[6,7]*, En Ma[8]*, Zhi-Wei Shan[1]*

The PDF file includes:

**Supplementary Figures**

Supplementary Figure 1: Summary of stress-strain curves of 11 submicron Mg pillars showing large plastic strains in c-axis compression

Supplementary Figure 2: Characterization of the 13 grains in the flattened sample by using SADP and dark-field TEM imaging

Supplementary Figure 3: High resolution TEM images showing the grain boundary structures

Supplementary Figure 4: Another example showing a Mg single crystal pillar flattened under c-axis compression, transforming into ultrafine grains in a strain burst

Supplementary Figure 5: MD simulation showing the formation of new grains during c-axis compression of a Mg single crystal

Supplementary Figure 6: Schematics showing the differences between $\{10\bar{1}1\}$ deformation twinning and Py-B transformation in terms of crystallography

**Supplementary Tables**

Supplementary Table 1: The Schmid factors for various slip and twinning systems under c-axis compression

Supplementary Table 2: Sample dimensions and mechanical data

Supplementary Table 3: Misorientations between grains in Figure 2a and Supplementary Figures 3 and 4

Supplementary Table 4: Estimation of the strain produced by different plastic carriers in the pillar shown in Figure 2

**Supplementary Figure 1. Summary of stress-strain curves of 11 submicron Mg pillars showing large plastic strains in *c*-axis compression.** Sample dimensions and corresponding mechanical data are listed in Supplementary Table 2. Scale bars, 200 nm. Insets, TEM and SEM images before and after tests showing the shape changes. To minimize the potential temperature-rise effect during mechanical loading, in some pillars, the compression loads were purposely stopped then reloaded after waiting for about 5 minutes (P8 once, P3 and P7 twice). In this case, the heat accumulated during plastic deformation would have enough time to transfer from the severely deformed area to the substrate area. The top view of the flattened sample P8 is provided, indicating that the pancaking is transverse anisotropic.

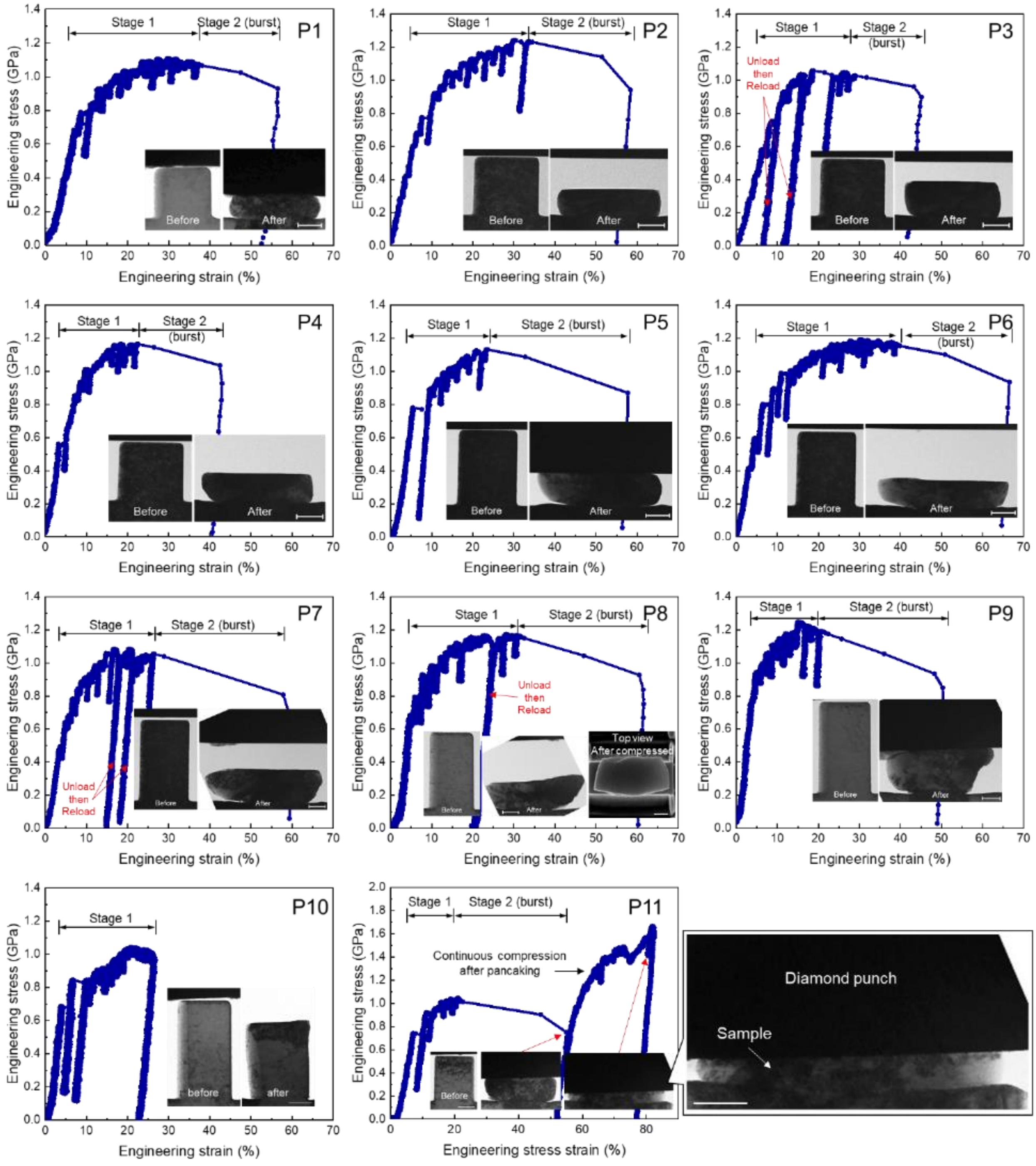

**Supplementary Figure 2. Characterization of the 13 grains in the flattened sample by using SADP and dark-field TEM imaging. a** Bright-field image of the flattened sample. **b** DPs acquired from the region that contains grains 3, 8, 10 and 11 and the corresponding dark-field TEM images. When using $\vec{g} = \{0001\}_{grain\ 11}$, grains 0, 1 and 11 are bright, indicating grains 1 and 11 are both residual matrix because grain 0 is matrix. When using $\vec{g} = \{0001\}_{grain\ 8}$, grains 6 and 9 are bright, indicating they have similar orientation with grain 8. **c** DPs acquired from the region at grain 4 and the corresponding dark-field TEM images. When using $\vec{g} = \{0002\}_{grain\ 4}$, grains 5 and 7 are bright, indicating they have similar orientation with grain 4. **d** DPs acquired from the region at grain 12 and the corresponding dark-field TEM images. When using $\vec{g} = \{0004\}_{grain\ 12}$, grain 2 is bright, indicating it has similar orientation with grain 12.

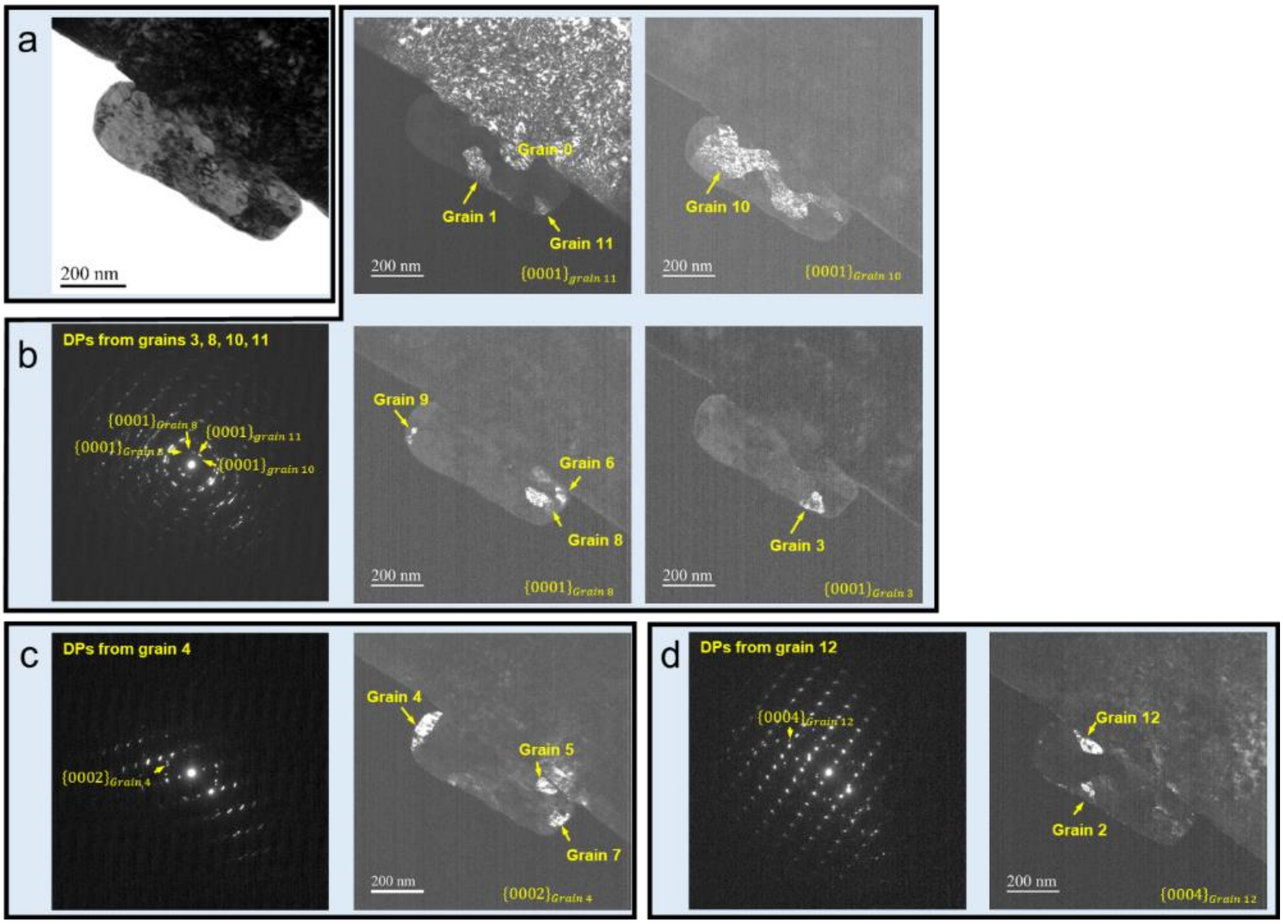

**Supplementary Figure 3. High resolution TEM images showing the grain boundary structures.** **a** The central image is constructed from a series of high resolution TEM images. Grain boundaries are outlined by the red dashed lines. The red arrow indicates the *c*-axis of each grain. The surrounding high resolution TEM images show typical grain boundary structures (marked by the white dashed lines). The grain boundaries are mainly composed of $\{0002\}/\{10\bar{1}0\}$, $\{0002\}/\{10\bar{1}1\}$, $\{0002\}/\{10\bar{1}3\}$, $\{10\bar{1}0\}/\{10\bar{1}1\}$ and $\{10\bar{1}0\}/\{10\bar{1}3\}$ interfaces. **b** Two low-angle grain boundaries in grain 10, which divide grain 10 into smaller parts 10-a,10-b,10-c.

a

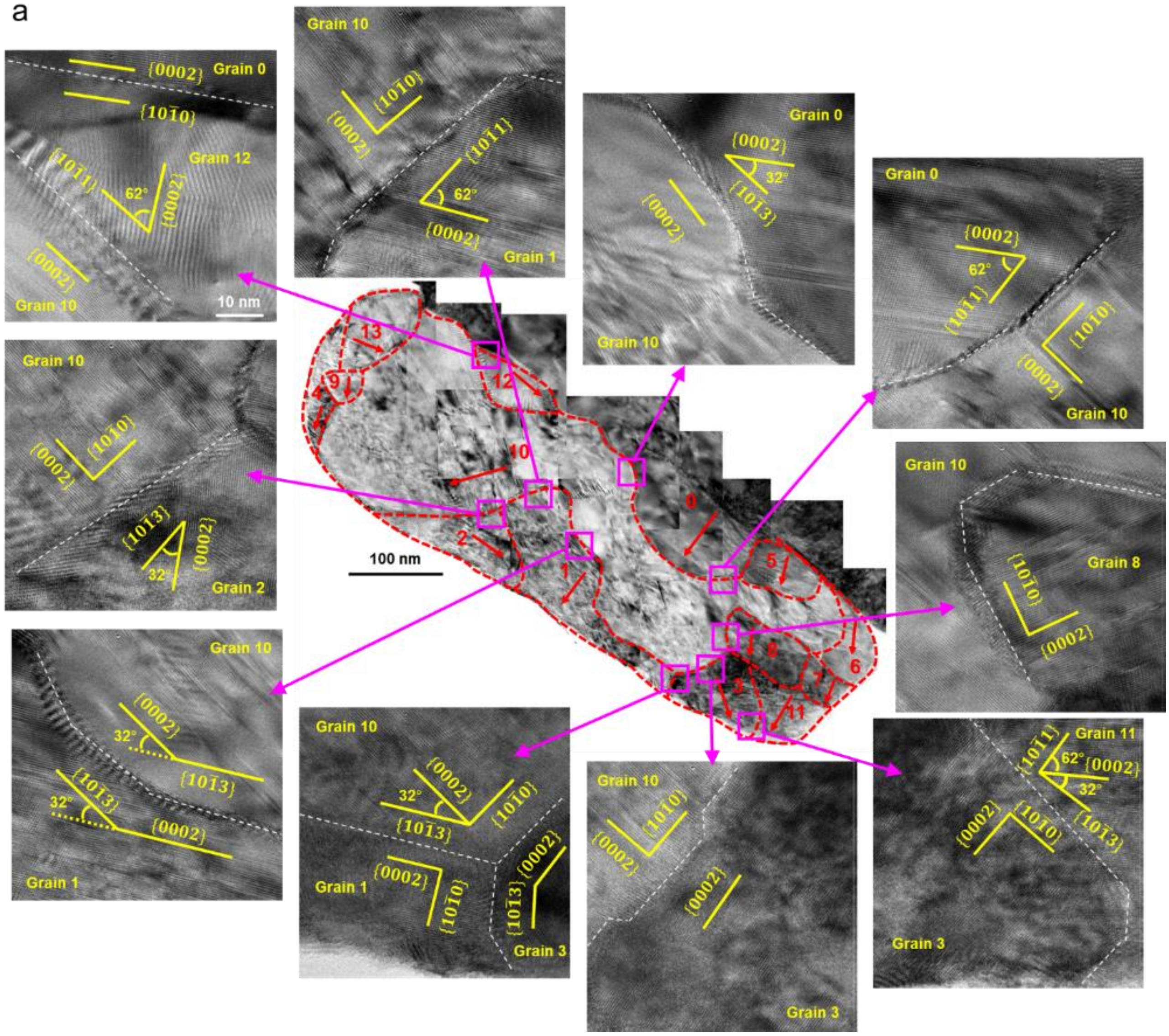

b

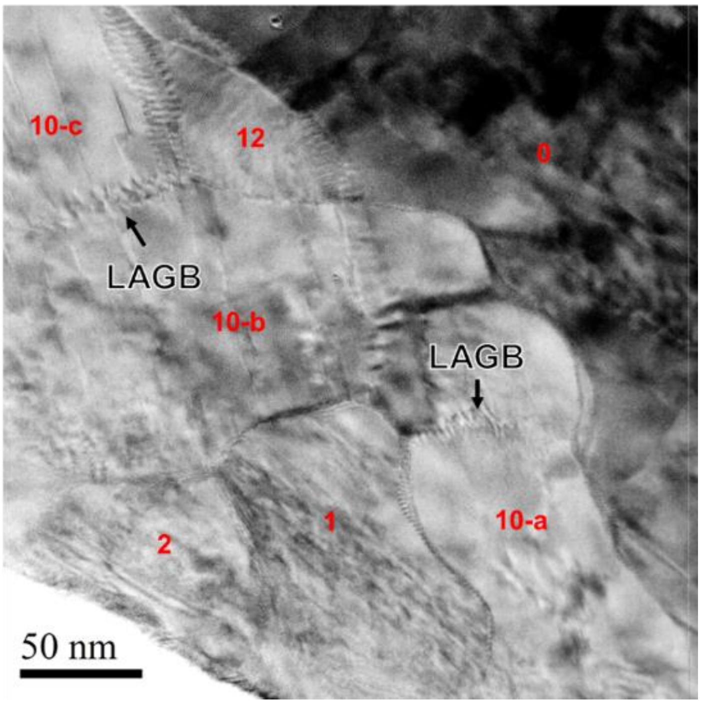

**Supplementary Figure 4. Another example showing a Mg single crystal pillar flattened under *c*-axis compression, transforming into ultrafine grains in a strain burst. a** Snapshots from in-situ movie. Viewing direction, $\sim\langle 2\bar{1}\bar{1}0\rangle$. **b** Corresponding stress-strain curve. **c** Ultrafine grains in the flattened sample. White arrow indicates the *c*-axis. The black contrast around the sample comes from Pt protection layer. Inset, SADP acquired from the entire sample. **d** SADPs acquired from each grain. Zone axis, $\sim\langle 2\bar{1}\bar{1}0\rangle$. The $\{0002\}$ spots are marked by white cycles.

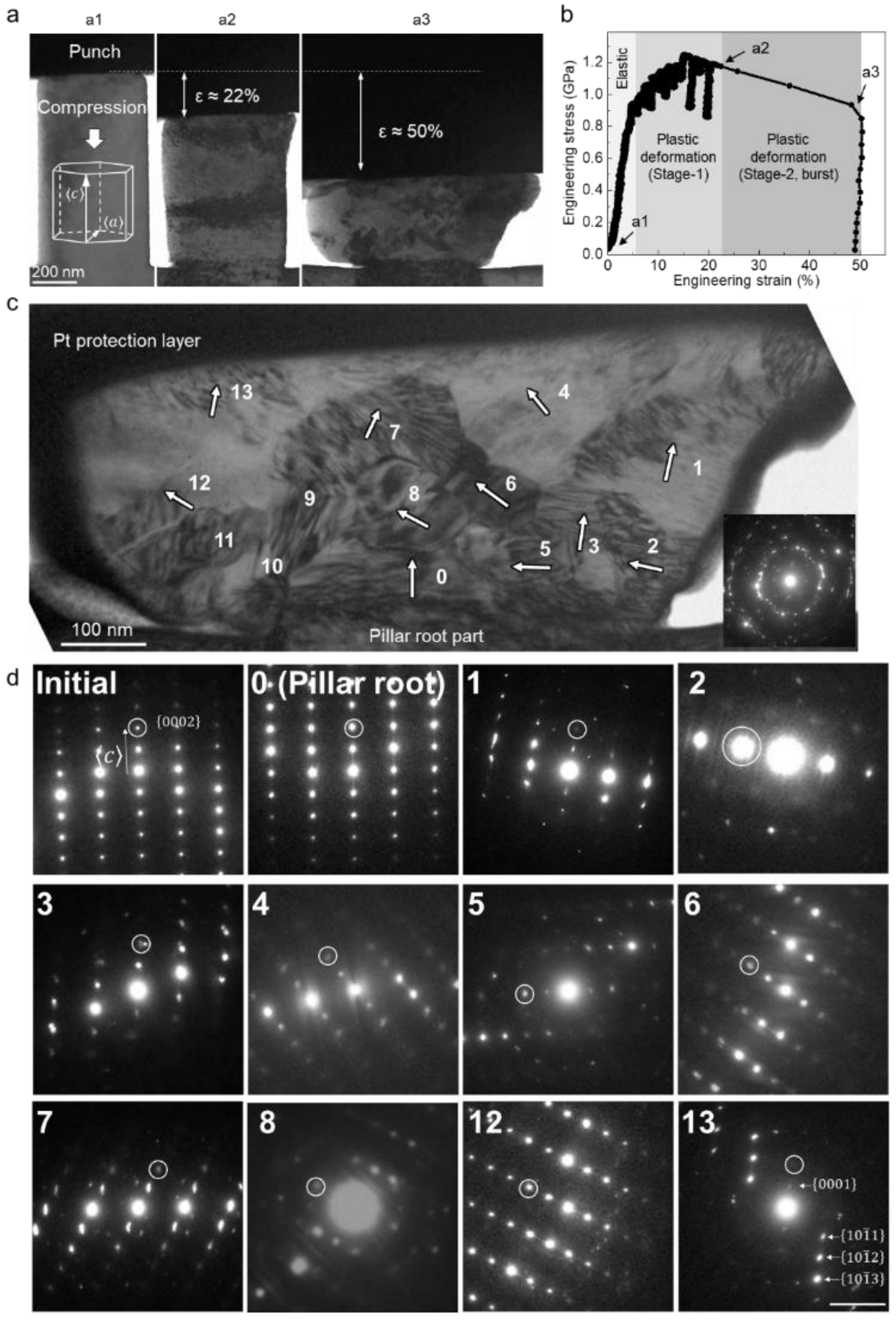

**Supplementary Figure 5. MD simulation showing the formation of new grains during *c*-axis compression of a Mg single crystal.** The basal planes are colored in red and blue in matrix to represent the …*ABAB*… stacking sequence in HCP structure to better identify the lattice transformation. **a** 4 new grains formed during deformation. Magnified image of the Py/B interfaces is shown in right. The $\langle 2\bar{1}\bar{1}0\rangle$ is co-shared by both matrix and new grain 1. The orientation relationship between new grain 1 and matrix satisfies $\{0002\}_{New\ grain\ 1} \parallel \{10\bar{1}1\}_{Matrix}$, and $\langle 10\bar{1}0\rangle_{New\ grain\ 1} \parallel \langle 10\bar{1}\bar{2}\rangle_{Matrix}$. **b, c** The growth of grain 1 via migration of Py/B and $\{10\bar{1}0\}/\{10\bar{1}3\}$ interfaces. The latter type of interface is experimentally observed as shown in Figure 5.

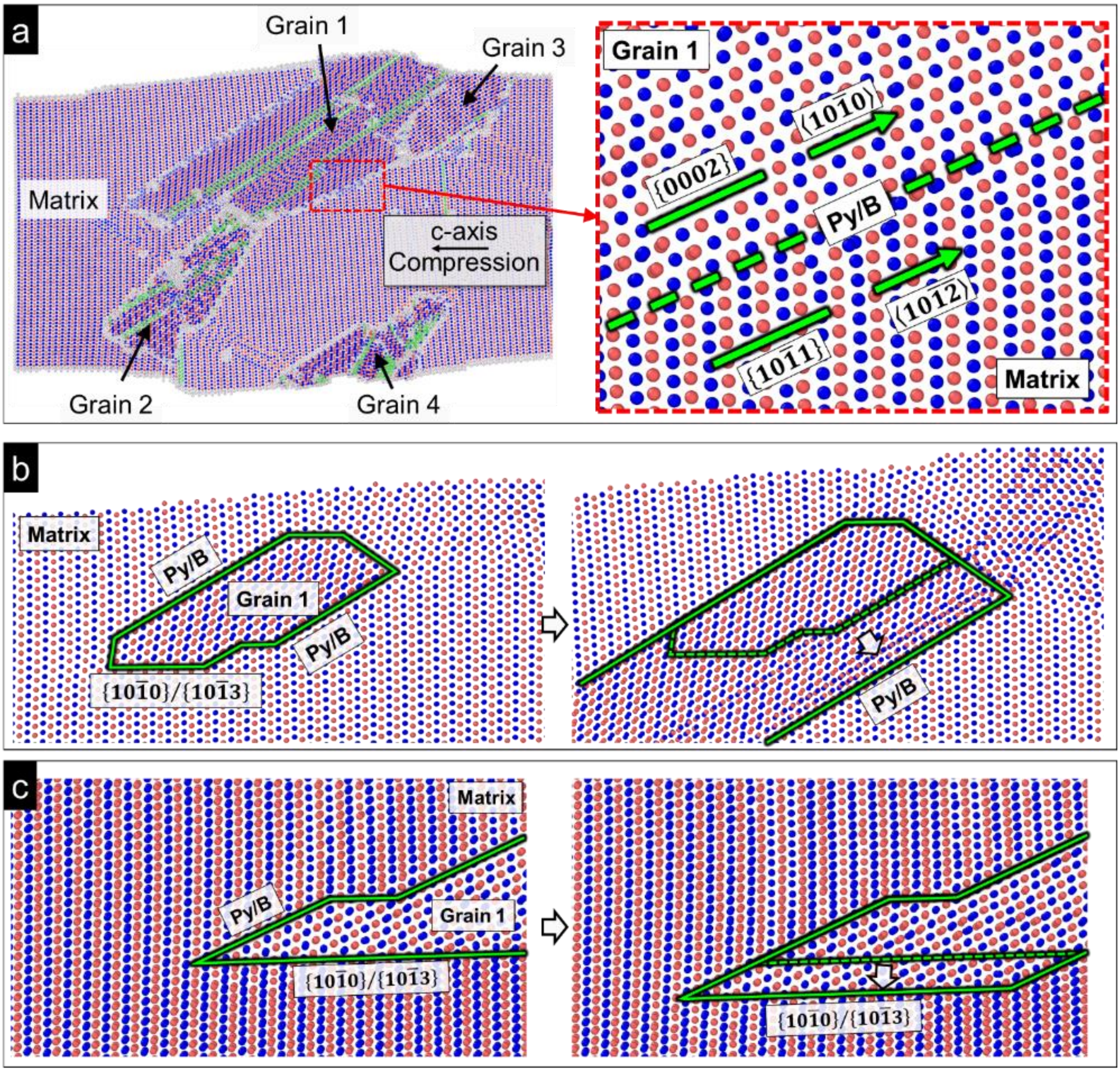

**Supplementary Figure 6. Schematics showing the differences between $\{10\bar{1}1\}$ deformation twinning and Py-B transformation in terms of crystallography.** Viewing direction, $\langle 2\bar{1}\bar{1}0\rangle$. **a** In $\{10\bar{1}1\}$ deformation twinning, the twinned lattice is symmetrical to the matrix lattice about the twin boundary (TB). The accurate angle between TB and basal planes in matrix is 62°, the same as between TB and basal planes in twin lattice. One set of the $\{10\bar{1}1\}$ planes is the invariant plane (twinning plane), co-shared by both matrix lattice and twin lattice. The TB is parallel to this $\{10\bar{1}1\}$ twinning plane. The accurate angle between the *c*-axis in matrix and in twin is 56°, i.e. a $56°\langle 2\bar{1}\bar{1}0\rangle$ orientation relationship. **b** In Py-B transformation, the boundary is Py/B interface. The lattice in matrix and new grain is not symmetrical about this interface. The accurate angle between the *c*-axis in matrix and in new grain is 62°, i.e. a $62°\langle 2\bar{1}\bar{1}0\rangle$ orientation relationship.

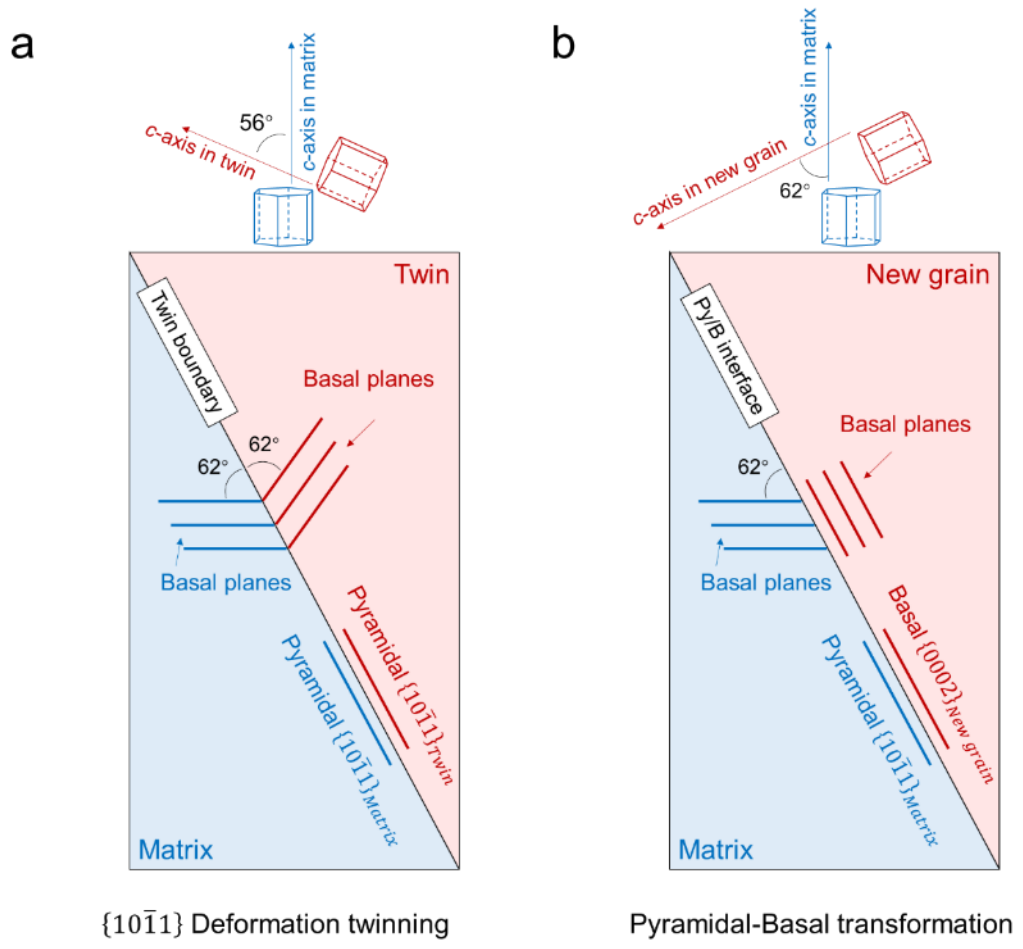

**Supplementary Table 1. The Schmid factors for various slip and twinning systems under *c*-axis compression.** The possibly activated deformation modes are pyramidal $\langle c+a \rangle$ slip and contraction twinning. The latter was not observed in the current experiments.

| Slip and twinning systems | Schmid factors | Calculated CRSS at the yield point in Figure 1g ($\sigma_y$=*680 MPa*) |
|---|---|---|
| $\{0002\}\langle 2\bar{1}\bar{1}0\rangle$ (basal <a> slip) | ~0 | - |
| $\{10\bar{1}0\}\langle 2\bar{1}\bar{1}0\rangle$ (prismatic <a> slip) | ~0 | - |
| $\{10\bar{1}0\}\langle 0001\rangle$ (prismatic <c> slip) | ~0 | - |
| $\{10\bar{1}1\}\langle 2\bar{1}\bar{1}0\rangle$ (pyramidal I <a> slip) | ~0 | - |
| $\{10\bar{1}1\}\langle 2\bar{1}\bar{1}3\rangle$ (pyramidal I <c+a> slip) | ~0.4 | 272 |
| $\{11\bar{2}2\}\langle 2\bar{1}\bar{1}3\rangle$ (pyramidal II <c+a> slip) | ~0.45 | 306 |
| $\{10\bar{1}1\}\langle 10\bar{1}\bar{2}\rangle$ (contraction twinning) | ~0.41 | - |
| $\{10\bar{1}2\}\langle 10\bar{1}\bar{1}\rangle$ (extension twinning) | ~-0.5 | - |

**Supplementary Table 2. Sample dimensions and mechanical data.** The P1 pillar is used in Figures 1, 2 and 7, Supplementary Figure 2 and 3 and Supplementary Movie 1. The P8 pillar is used in Figure 3. The P10 pillar is used in Figure 4. The compression test of this pillar was purposely stopped after the new grain appeared. The P9 pillar is used in Supplementary Figure 4. The P11 pillar is used in Supplementary Movie 2.

| #No. | Width × thickness × height (nm) | Stress before burst (GPa) | Strain (elastic) (%) | Strain (Stage-1) (%) | Strain (Stage-2) (%) | Maximum strain (%) |
|---|---|---|---|---|---|---|
| **P1** | 417 × 425 × 409 | 1.07 | 6.9 | 29.8 | 19.7 | 56.4 |
| **P2** | 453 × 437 × 427 | 1.23 | 7.6 | 26.8 | 24.0 | 58.4 |
| **P3** | 451 × 457 × 431 | 1.02 | 6.3 | 23.0 | 15.5 | 44.8 |
| **P4** | 453 × 419 × 434 | 1.16 | 3.2 | 19.0 | 20.8 | 43.0 |
| **P5** | 453 × 437 × 616 | 1.13 | 5.4 | 18.2 | 34.1 | 57.7 |
| **P6** | 453 × 454 × 616 | 1.16 | 6.3 | 33.1 | 26.7 | 66.1 |
| **P7** | 454 × 437 × 803 | 1.00 | 6.6 | 20.1 | 34.0 | 60.7 |
| **P8** | 414 × 427 × 823 | 1.16 | 5.0 | 26.2 | 30.4 | 61.6 |
| **P9** | 415 × 407 × 830 | 1.17 | 4.8 | 18.5 | 26.9 | 50.2 |
| **P10** | 431 × 456 × 878 | 0.98 (before unloading) | 3.9 | 22.6 (purposely unloaded) | - | 26.5 |
| **P11** | 431 × 441 × 524 | 1.02 | 7.1 | 15.5 | 32.3 | 81.9 (including the continuous compression) |

**Supplementary Table 3. Misorientations between grains in Figure 2a and Supplementary Figures 3 and 4.** The misorientation angles were measured by the angle between the *c*-axis of each grain. The zone axis is $\langle 2\bar{1}\bar{1}0 \rangle$.

| Figures 2a and Supplementary Figures 3 | | |
|---|---|---|
| **Misorientation with respect to $\langle 2\bar{1}\bar{1}0 \rangle$** | | **Possible forming path** |
| *grain 0* | - | Matrix |
| *grain 1 / matrix* | 2° | Residual matrix |
| *grain 11 / matrix* | 7° | Residual matrix |
| *grain 3 / grain 1* | 61° | PyB from *grain 1* |
| *grain 10 / grain 1* | 35° | PyB then $\{10\bar{1}2\}$ twinning, 180°-(62°+86°)=32° |
| *grain 6 / matrix* | 30° | PyB then $\{10\bar{1}2\}$ twinning, 180°-(62°+86°)=32° |
| *grain 8 / matrix* | 32° | PyB then $\{10\bar{1}2\}$ twinning, 180°-(62°+86°)=32° |
| *grain 9 / matrix* | 32° | PyB then $\{10\bar{1}2\}$ twinning, 180°-(62°+86°)=32° |
| *grain 4 / matrix* | 21° | PyB then $\{10\bar{1}2\}$ twinning, 86°-62°=24° |
| *grain 5 / matrix* | 23° | PyB then $\{10\bar{1}2\}$ twinning, 86°-62°=24° |
| *grain 7 / matrix* | 21° | PyB then $\{10\bar{1}2\}$ twinning, 86°-62°=24° |
| *grain 2 / grain 1* | 84° | $\{10\bar{1}2\}$ twinning from *grain 1* during unloading |
| *grain 12 / matrix* | 86° | $\{10\bar{1}2\}$ twinning from *grain 0* during unloading |
| *grain 13 / grain 4* | 84° | $\{10\bar{1}2\}$ twinning from *grain 4* during unloading |

| Supplementary Figure 4 | | |
|---|---|---|
| **Misorientation with respect to $\langle 2\bar{1}\bar{1}0 \rangle$** | | **Possible forming path** |
| *grain 0* | - | Matrix |
| *grain 1 / matrix* | < 10° | Residual matrix |
| *grain 3 / matrix* | < 6° | Residual matrix |
| *grain 13 / matrix* | < 10° | Residual matrix |
| *grain 6 / matrix* | ~ 62° | PyB from matrix |
| *grain 8 / matrix* | ~ 62° | PyB from matrix |
| *grain 12 / matrix* | ~ 62° | PyB from matrix |
| *grain 4 / grain 7* | ~ 66° | PyB from *grain 7* |
| *grain 7 / matrix* | ~ 27° | PyB then $\{10\bar{1}2\}$ twinning, 86°-62°=24° |
| *grain 2 / matrix* | ~ 90° | $\{10\bar{1}2\}$ twinning from matrix during unloading |
| *grain 5 / matrix* | ~ 89° | $\{10\bar{1}2\}$ twinning from matrix during unloading |
| *grains 9, 10 and 11* | - | Far from a low index zone axis under current viewing |

**Supplementary Table 4. Estimation of the strain produced by different plastic carriers in the pillar shown in Figure 2.** The calculation procedure is described in Methods section. The twins in grains 2, 12 and 13 are likely formed in unloading, so they are not counted in. The total strain produced in Stage-2 is 19.7% (Supplementary Table 2-P1). Therefore, the strain produced by dislocations slip in Stage-2 deformation, $\varepsilon_{dislocation}$ is 12% (19.7%-3.85%-3.85%=12%).

| Grain | v | $\varepsilon_{Py-B}$ | $\varepsilon_{twin}$ |
|---|---|---|---|
| 0 | 8.81% | 0 | 0 |
| 1 | 10.52% | 0 | 0 |
| 2 | 5.06% | 0 | 0 |
| 3 | 4.39% | 0.26% | 0 |
| 4 | 2.40% | 0.14% | 0.15% |
| 5 | 1.74% | 0.10% | 0.11% |
| 6 | 2.15% | 0.13% | 0.13% |
| 7 | 1.37% | 0.08% | 0.09% |
| 8 | 3.15% | 0.18% | 0.20% |
| 9 | 0.87% | 0.05% | 0.05% |
| 10 | 49.68% | 2.91% | 3.12% |
| 11 | 3.29% | 0 | 0 |
| 12 | 2.51% | 0 | 0 |
| 13 | 4.07% | 0 | 0 |
| Total | 100% | $\sum \varepsilon_{Py-B}$ =3.85% | $\sum \varepsilon_{twin}$=3.85% |
