# Supplementary material for: Rejuvenation of plasticity via deformation graining in magnesium
Source: Nat Commun. 2022 Feb 25;13:1060. doi: 10.1038/s41467-022-28688-9 (PMC8881527; doi:10.1038/s41467-022-28688-9)
Supplement: Supplementary file 3 — Description of Additional Supplementary Files [file 41467_2022_28688_MOESM3_ESM.pdf]

Description of Additional Supplementary Files

File name: Supplementary Movie 1

Description: The two-stage plastic deformation

File name: Supplementary Movie 2

Description: Rejuvenated dislocation activities in new grains
